# The high molecular weight dipeptidyl peptidase IV Pol d 3 is a major allergen of *Polistes dominula* venom

**DOI:** 10.1038/s41598-018-19666-7

**Published:** 2018-01-22

**Authors:** Maximilian Schiener, Christiane Hilger, Bernadette Eberlein, Mariona Pascal, Annette Kuehn, Dominique Revets, Sébastien Planchon, Gunilla Pietsch, Pilar Serrano, Carmen Moreno-Aguilar, Federico de la Roca, Tilo Biedermann, Ulf Darsow, Carsten B. Schmidt-Weber, Markus Ollert, Simon Blank

**Affiliations:** 1 Center of Allergy and Environment (ZAUM), Technical University of Munich and Helmholtz Center Munich, Member of the German Center of Lung Research (DZL), Munich, Germany; 2 Department of Infection and Immunity, Luxembourg Institute of Health (LIH), Esch-sur-Alzette, Luxembourg; 3 Department of Dermatology and Allergy Biederstein, Technical University of Munich, Munich, Germany; 4 Immunology Department, CDB Hospital Clinic de Barcelona, Universitat de Barcelona, Barcelona, Spain; 5 Department of Environmental Research and Innovation, Luxembourg Institute of Science and Technology, Belvaux, Luxembourg; 6 Maimonides Institute for Research in Biomedicine (IMIBIC), Córdoba, Spain; 7 Hospital Universitario Reina Sofía, Córdoba, Spain; 8 Allergy Unit, Pneumology Department, ICR, Hospital Clinic de Barcelona, Barcelona, Spain; 9 Department of Dermatology and Allergy Center, Odense Research Center for Anaphylaxis, University of Southern Denmark, Odense, Denmark

## Abstract

Hymenoptera venom allergy can cause severe anaphylaxis in untreated patients. *Polistes dominula* is an important elicitor of venom allergy in Southern Europe as well as in the United States. Due to its increased spreading to more moderate climate zones, *Polistes* venom allergy is likely to gain importance also in these areas. So far, only few allergens of *Polistes dominula* venom were identified as basis for component-resolved diagnostics. Therefore, this study aimed to broaden the available panel of important *Polistes* venom allergens. The 100 kDa allergen Pol d 3 was identified by mass spectrometry and found to be a dipeptidyl peptidase IV. Recombinantly produced Pol d 3 exhibited sIgE-reactivity with approximately 66% of *Polistes* venom-sensitized patients. Moreover, its clinical relevance was supported by the potent activation of basophils from allergic patients. Cross-reactivity with the dipeptidyl peptidases IV from honeybee and yellow jacket venom suggests the presence of exclusive as well as conserved IgE epitopes. The obtained data suggest a pivotal role of Pol d 3 as sensitizing component of *Polistes* venom, thus supporting its status as a major allergen of clinical relevance. Therefore, Pol d 3 might become a key element for proper diagnosis of *Polistes* venom allergy.

## Introduction

Stings of hymenoptera of different species can cause life-threatening IgE-mediated anaphylaxis in venom-allergic patients. The most prominent elicitors of venom allergy in Western and Central Europe are honeybees (*Apis mellifera*) and yellow jackets (*Vespula vulgaris*)^1^. Additionally, allergic reactions to paper wasps, especially to *Polistes dominula*, are common in Southern Europe and the Unites States^2–6^. *Polistes dominula*, known to be domestic in Southern Europe, is an invasive species entering the US (1970s) from the north-east to the west coast (1990s)^7^, South Africa (2008)^8^ and central Europe (1956)^9^. Therefore, allergy to *Polistes dominula* venom (PDV) will most probably gain importance also in other areas.

The only curative treatment for venom allergy is venom-specific immunotherapy (VIT)^10,11^. To ensure a successful treatment and to avoid the increased risk of side effects, possible *de novo* sensitizations and higher costs, the correct therapeutic venom must be selected^4,12^. To accomplish this, a careful anamnesis is important to identify the insect that elicited the allergic reaction. Due to the number and hard to discriminate phenotypes of insects that can induce allergic reactions, many patients and allergy specialists are not able to correctly distinguish between different hymenoptera species such as *Vespula spp*. and *Polistes spp*.^13^. Therefore, clinicians depend on additional diagnostic tests.

The increased knowledge of the composition of hymenoptera venoms has led to major improvements in diagnostic approaches and created the field of molecular or component-resolved diagnostics (CRD) in hymenoptera venom allergy^4,14,15^. In combination with skin testing and venom extract-based specific IgE (sIgE) diagnostics, CRD has created added clinical value for a proper allergy diagnosis. For CRD of hymenoptera venom allergy commercial allergens are available for different test platforms to determine sIgE serum titers^6^.

For the diagnosis and discrimination of honeybee venom (HBV)- and yellow jacked venom (YJV)-allergic patients, many commercial allergens are available, allowing for high diagnostic sensitivity and specificity^16–22^. However, for the diagnosis of PDV allergy only one allergen (antigen 5, Pol d 5) is commercially available. Furthermore, only three allergens of PDV have been identified in the past, namely phospholipase A1 (Pol d 1)^23^, protease (Pol d 4)^24^ and antigen 5 (Pol d 5)^25^ from which Pol d 4 is a minor allergen with restricted diagnostic importance (unpublished data). Even though, we have recently shown, that *Polistes* venom is free of cross-reactive carbohydrate determinant-(CCD-)based cross-reactivity^26^, available extract-based diagnostic approaches to discriminate between PDV und YJV allergy are hampered by extensive protein cross-reactivity^2,3,27,28^.

An increased knowledge of the allergen composition of PDV and the availability of further important PDV components are likely to generate added clinical benefit for proper and advanced diagnostics. Therefore, our study aimed to identify and immunologically characterise additional major allergens of PDV. The here described allergen Pol d 3 is a homologue of the prominent dipeptidyl peptidase IV (DPP IV) allergens Api m 5 and Ves v 3 from HBV and YJV^29^. Extensive sIgE reactivity and the ability to activate basophils from allergic patients clearly support the role of Pol d 3 as major allergen of PDV as well as its potential to be a key element for molecular diagnostic approaches.

## Results

### Identification of Pol d 3

**Figure 1:**
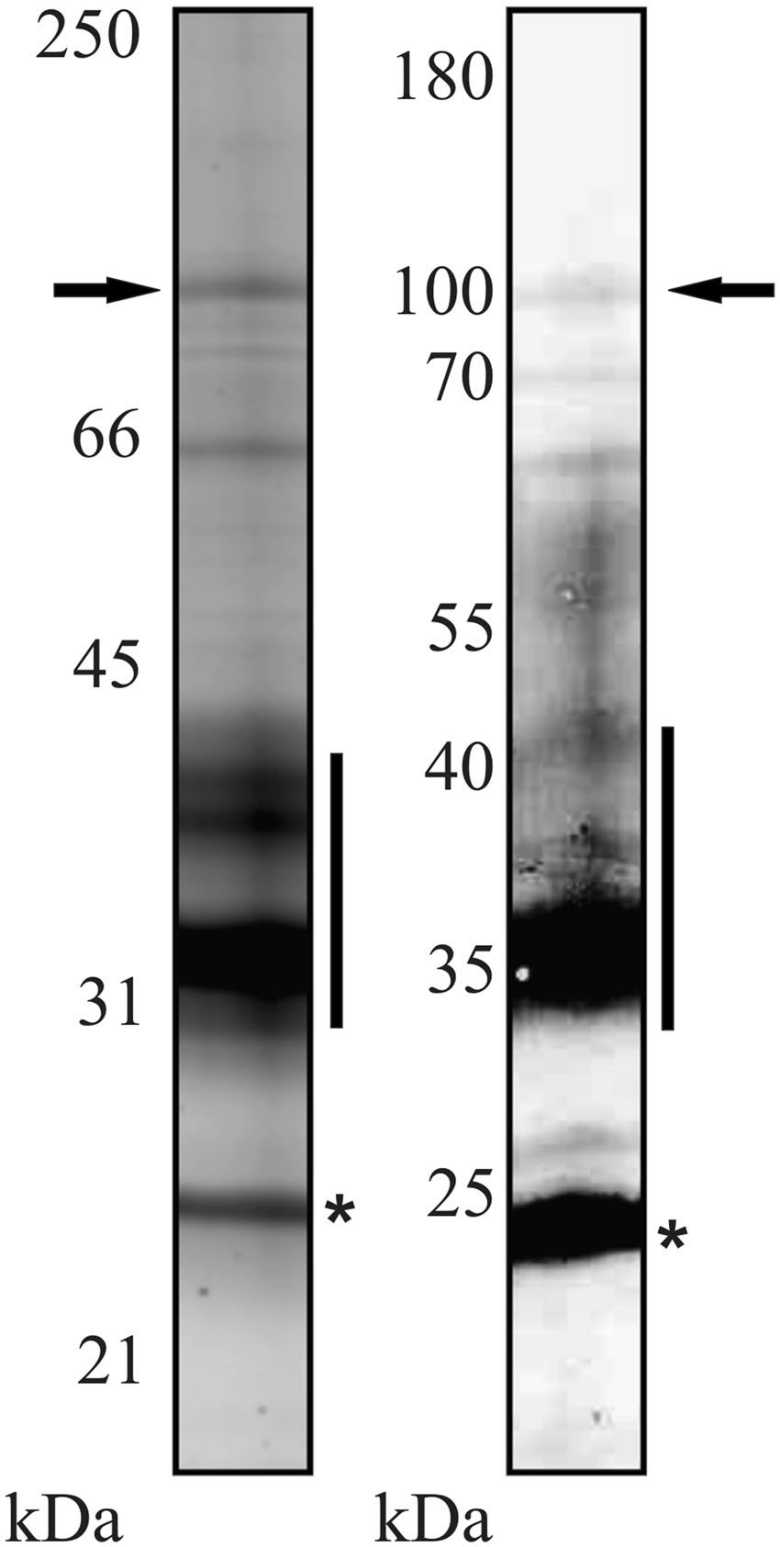
Detection of Pol d 3 in *Polistes dominula* venom. SDS-PAGE and protein staining (SyproRuby staining) of PDV (left) and sIgE-immunoreactivity of pooled sera from PDV-allergic patients with PDV in Western blot (right). The arrow indicates the 100 kDa band that was subjected to tandem mass spectrometry and MALDI-TOF analyses. Asterisks indicate the band that was identified as Pol d 5 and black bars the area of the gel/blot in which Pol 1 and Pol d 4 were identified. Shown are parts of the gel. Blot and full-length gels are shown in Supplemental Fig. S3. The kDa values correspond to the protein marker (not shown) which can be found in Fig. S3.

**Figure 2:**
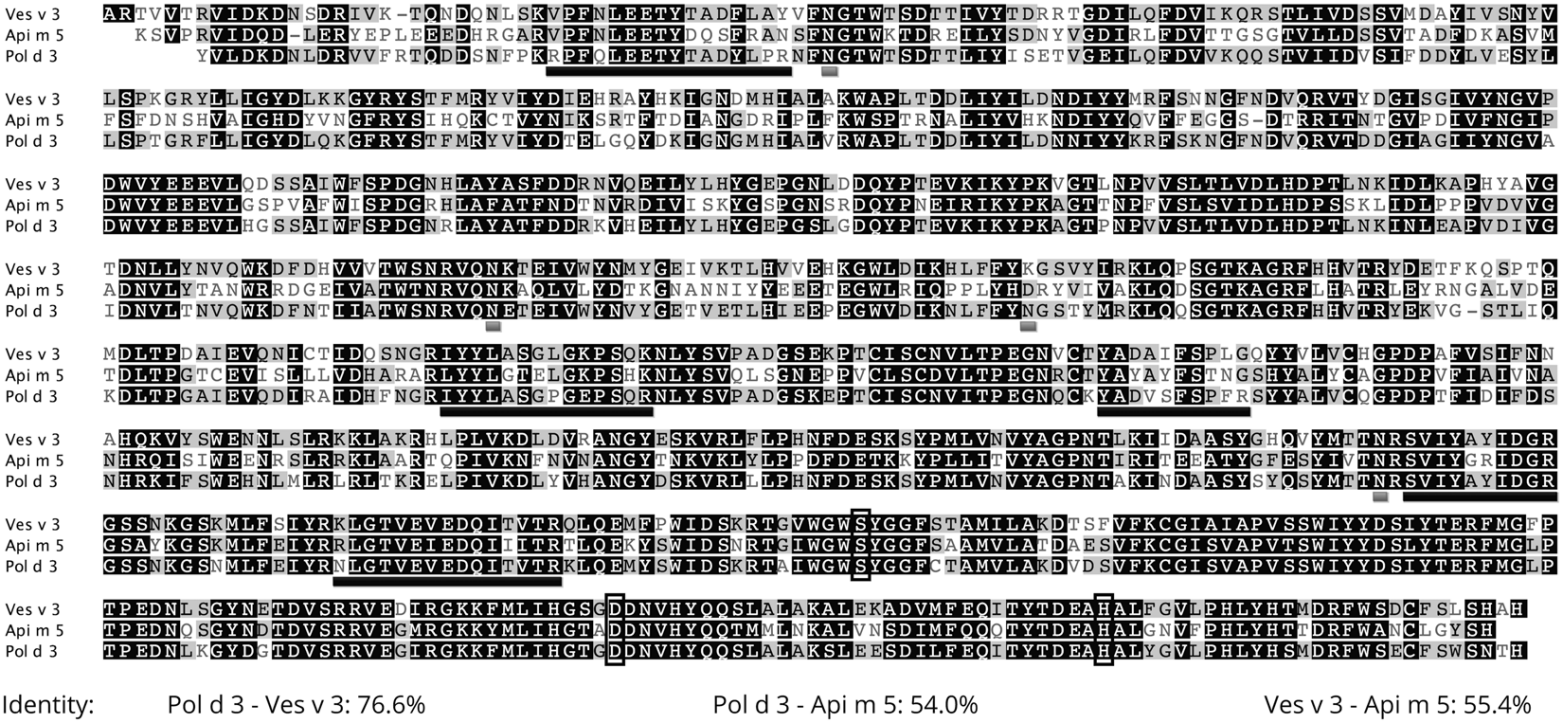
Alignment of Pol d 3 with Ves v 3 and Api m 5. Mature amino acid sequences of Pol d 3 (*Polistes dominula*), Ves v 3 (*Vespula vulgaris*) and Api m 5 (*Apis mellifera*) are shown. Black shaded amino acids are identical between all three proteins, gray shaded amino acids are shared by two proteins and amino acids not shaded are unique to the individual protein. Peptides identified by tandem mass spectrometry are underlined in black and potential N-glycosylation sites in gray. The residues involved in the conserved active center of the enzymes are represented boxed. Overall amino acid identity between the different proteins is stated in percent.

Immunoblots using PDV and pooled sera of PDV-allergic patients (n = 5) revealed sIgE reactivity with several venom components (Fig. 1). Prominent bands at approximately 23 and 31 to 40 kDa were identified by mass spectrometry as the known allergens antigen 5 (Pol d 5), protease (Pol d 4) and phospholipase A1 (Pol d 1), respectively. Moreover, prominent sIgE reactivity was obtained with a high molecular weight protein of approximately 100 kDa. The protein was subjected to *de novo* sequencing by tandem mass spectrometry. The five identified peptides (Fig. 2) yielded hits in a database search of predicted proteins coded by the recently published genome of *Polistes dominula*^30^ and identified the sIgE-reactive protein as venom dipeptidyl peptidase IV (GenBank accession XP_015174445). An additional matrix-assisted laser desorption ionization-time of flight (MALDI-TOF) analysis of the protein band then led to a sequence coverage of approximately 44% with the predicted sequence.

The nucleotide sequence (GenBank accession XM 015318959) of the newly identified allergen codes a mature protein of 751 amino acids and a calculated molecular weight of 86.2 kDa. The discrepancy between the calculated and the apparent molecular mass most likely is due to posttranslational modification by glycosylation as suggested by the presence of 4 or 3 N-linked glycosylation sites based on the binary profile or on average surface accessibility, respectively^31^. The protein belongs to the dipeptidyl peptidase IV superfamily, known to cleave dipeptides from the N-terminus of peptidic substrates, including many chemokines, neuropeptides, peptide hormones and venom peptides^32,33^. Therefore, it is a homologue of the well-established allergens Api m 5 (HBV) and Ves v 3 (YJV)^29^. The identity on protein level with Api m 5 and Ves v 3 is 54.0% and 76.6%, respectively. According to its allergenic properties and the homology to YJV Ves v 3 the new PDV allergen was assigned as Pol d 3.1010 in the WHO/IUIS Allergen Nomenclature Database^34^.

### Recombinant expression and characterisation of Pol d 3

**Figure 3:**
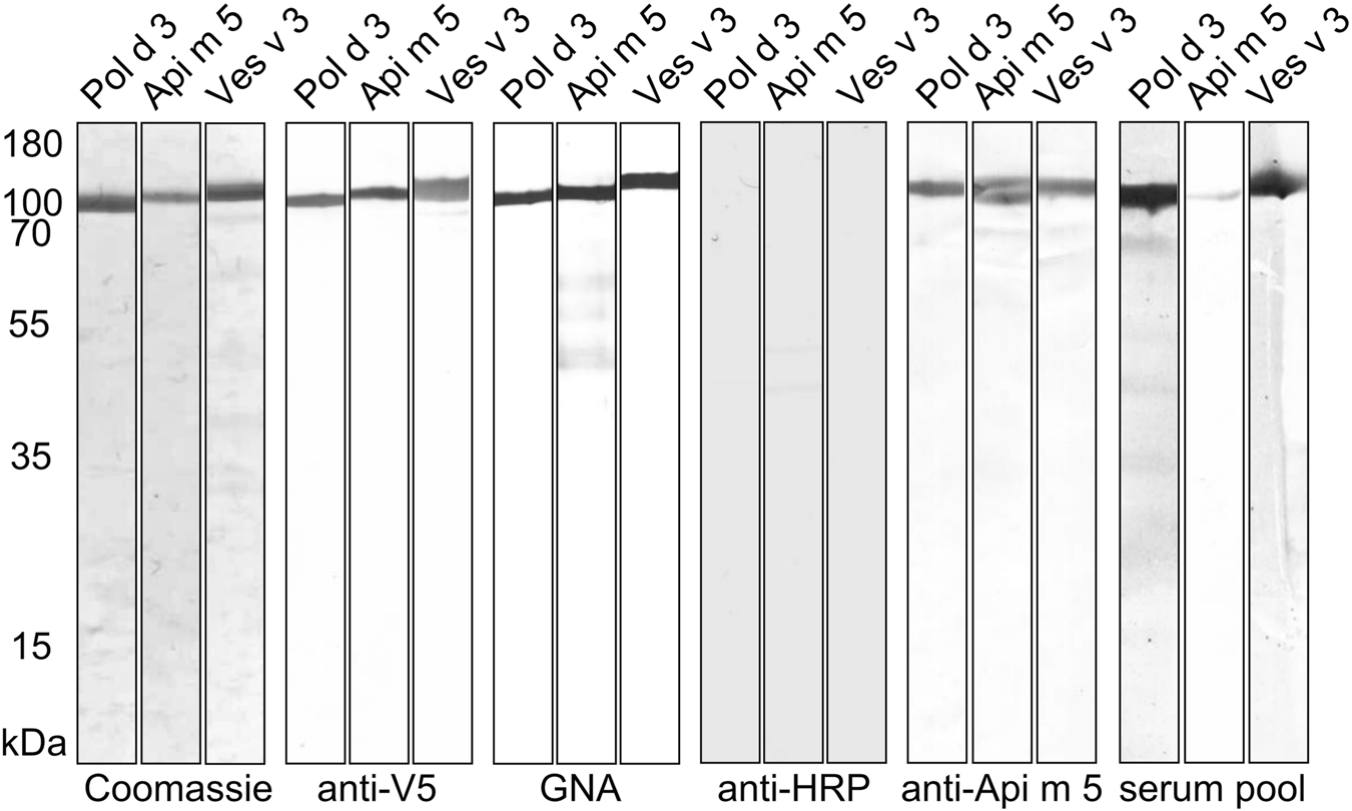
Recombinant expression and characterisation of Pol d 3. SDS-PAGE and Western blot analyses of Pol d 3 recombinantly produced in Sf9 insect cells in comparison with the HBV and YJV homologues Api m 5 and Ves v 3 either by Coomassie blue staining or anti-V5 epitope antibody, GNA (*Galanthus nivalis* agglutinin), anti-HRP antiserum, anti-Api m 5 antiserum and pooled sera of PDV-allergic patients. Shown are parts of one or more gels and blots and full-length gels and blots are given in Supplemental Fig. S3.

For recombinant expression, the coding region of Pol d 3 was amplified from PDV gland cDNA. Recombinant production was achieved by baculoviral infection of Sf9 insect cells and Ni^2+^-affinity chromatography yielded soluble recombinant Pol d 3 from culture supernatants with an apparent molecular weight of approximately 100 kDa as shown by Coomassie staining and reactivity with an antibody reacting with the V5-epitope tag added for recombinant expression (Fig. 3). Moreover, correct folding of the allergen was supported by enzymatic DPP IV activity (Supplementary Fig. S1) as well as by circular dichroism (CD) spectroscopy (Supplementary Fig. S2). For comparable analyses the homologous allergens from HBV and YJV, Api m 5 and Ves v 3, were produced as described previously^29^ (Fig. 3).

Reactivity with *Galanthus nivalis* agglutinin (GNA) (reactive with terminal 1,2-. 1,3- and 1,6-linked mannose residues) proves the presence of N-linked glycans (Fig. 3). However, staining with rabbit polyclonal horseradish peroxidase (HRP) antiserum, specific for α-1,3-core fucosylation, the structure responsible for CCD-based cross-reactivity, demonstrates the lack of CCDs (Fig. 3), as shown previously for other allergens produced in Sf9 insect cells^18,35–37^.

Furthermore, staining with a polyclonal rabbit Api m 5 antiserum shows reactivity with Api m 5 but also with Ves v 3 and Pol d 3, suggesting cross-reactivity between the three DPP IV hymenoptera allergens. Cross-reactivity was confirmed by sIgE-reactivity of pooled sera of PDV-allergic patients with all three allergens (Fig. 3).

### Activation of basophils from venom-allergic patients by Pol d 3

**Figure 4:**
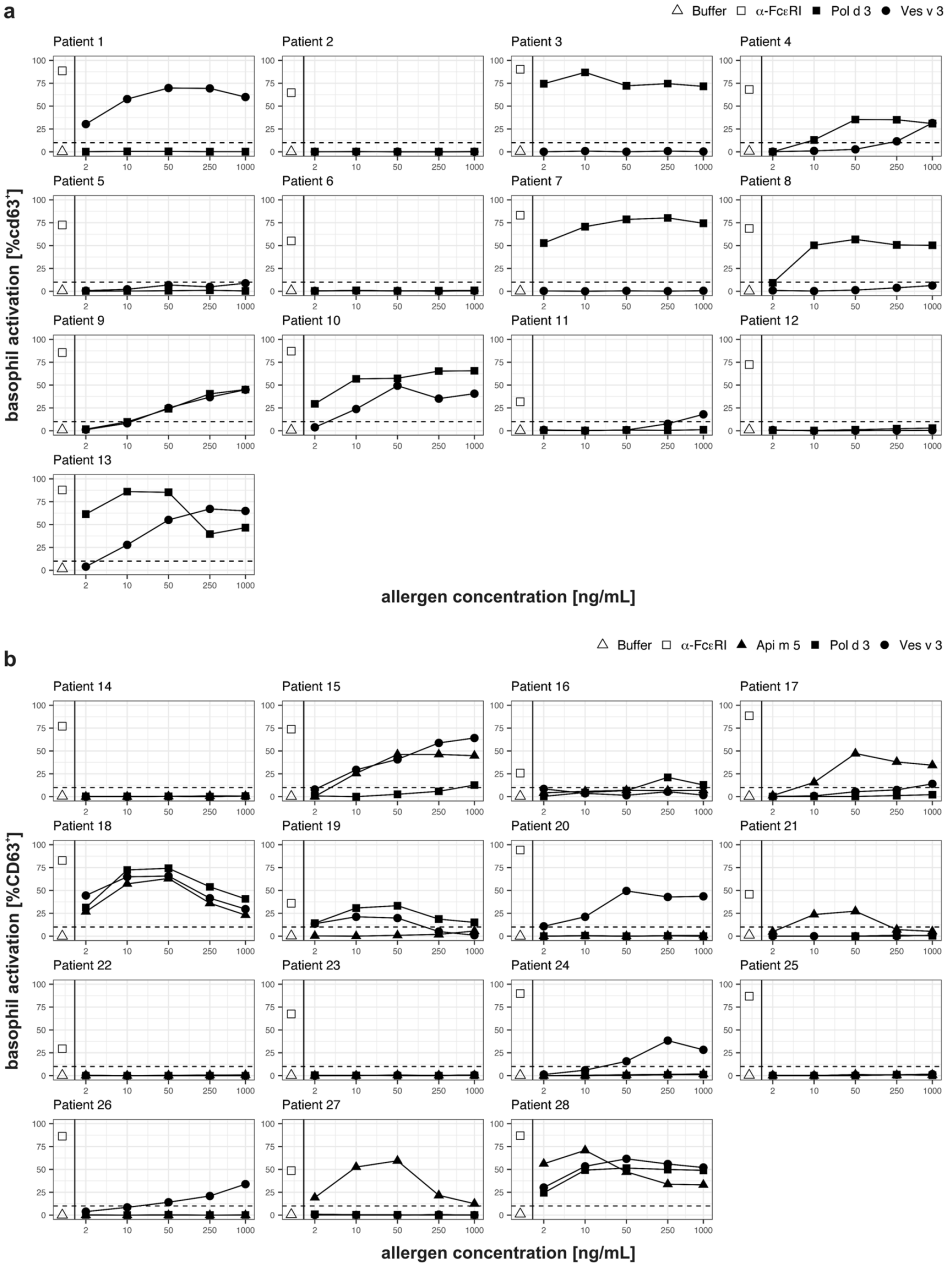
Basophil activation tests of (**a**) PDV- and/or YJV-allergic patients from Spain (area of Barcelona) and (**b**) HBV- and/or YJV-allergic patients from Germany with recombinant DPP IV allergens Pol d 3, Ves v 3 or Api m 5. Basophils were exposed to different concentrations of the DPP IV allergens. Additionally, stimulation with anti-FcεRI antibody (positive control) and plain stimulation buffer (negative control) is shown. Activation is shown as percentage of CD63^+^ out of total basophilic cells. The cut-off of the assay (10%) is represented as dotted line.

To address the capability of Pol d 3 to cross-link receptor-bound IgE and, thus, to activate effector cells, basophil activation tests (BATs) were performed (Fig. 4) as previously described^28,38^. First, 13 patients from Spain (from the area of Barcelona) with history of an allergic reaction to PDV and/or YJV were analysed for their reactivity with Pol d 3 and Ves v 3 (Fig. 4a). Three patients showed basophil activation only by Pol d 3 (patients 3, 7, 8), two only by Ves v 3 (patients 1, 11) and four by both allergens (patients 4, 9, 10, 13). Interestingly, for most of the patients these data nicely match the data of skin tests and/or sIgE levels to the venom extracts (i3, i77) and allergen components (i209, i210, i211) (Table 1). Therefore, for most patients BATs with the DPPs IV would have been able to identify the insect(s), the patient most likely shows primary sensitization to. For patient 4, showing stronger basophil activation by Pol d 3 compared to Ves v 3, but higher sIgE levels to YJV compared to PDV, clinical information is too scarce to identify the allergy-eliciting venom. Moreover, patient 13 exhibits basophil activation by both allergens but much higher sIgE level to PDV compared to YJV extract and a negative skin test to YJV. However, for this patient basophil activation by Pol d 3 is much stronger compared to Ves v 3, and Ves v 3-reactivity is most likely due to cross-reactivity. For patients 9 and 10 who show comparable activation patterns by both allergens, also clinical data suggest allergy to both venoms.

**Table 1:** Clinical data of patients analyzed in basophil activation test.

Patient ID	Skin test^1^ (i.c.) PDV	Skin test^1^ (i.c.) YJV	Skin test^1^ (i.c.) HBV	tIgE [kU/L]	sIgE PDV (i77) [kU_A_/L]	sIgE YJV (i3) [kU_A_/L]	sIgE HBV (i1) [kU_A_/L]	sIgE Pol d 5 (i210) [kU_A_/L]	sIgE Ves v 5 (i209) [kU_A_/L]	sIgE Ves v 1 (i211) [kU_A_/L]	sIgE Api m 1 (i208) [kU_A_/L]
1	neg.	0.001	neg.	55.5	n.d.	***4.32***	0.01	0.00	0.00	***5.16***	n.d.
2	1	1	neg.	237	***15.3***	***10.6***	0.02	***36.1***	***23.5***	0.07	0.00
3	0.1	0.1	neg.	12.3	***1.07***	***0.35***	0.02	***0.85***	**0.27**	**0.17**	0.00
4	n.d.	n.d.	n.d.	45.3	***0.59***	***1.78***	***2.81***	0.00	0.03	***1.75***	**0.18**
5	neg.	0.1	neg.	84	**0.30**	***1.54***	0.02	0.01	**0.12**	***1.58***	0.00
6	0.001	0.1	neg.	291	***15.9***	***3.53***	0.02	***7.88***	***5.53***	***2.16***	0.00
7	0.0001	0.1	neg.	305	***35.6***	***20.0***	**0.10**	***14.4***	***2.07***	***24.1***	0.00
8	0.01	0.1	0.1	168	***4.34***	***3.55***	***1.16***	***1.9***	***1.21***	***3.93***	***0.35***
9	0.1	0.01	neg.	53.5	***1.98***	***2.11***	0.02	***1*** ^***2***^	***1.2*** ^***2***^	n.d.	**<** **0.3** ^**2**^
10	0.01	0.0001	neg.	313	***42.3***	***22.4***	**0.16**	***17.5***	***9.76***	***22.8***	0.02
11	n.d.	n.d.	n.d.	257	**0.14**	***0.88***	0.08	0.00	***0.89***	0.00	0.00
12	1	neg.	neg.	74.6	**0.18**	**0.27**	0.00	**0.23**	**0.18**	0.08	0.00
13	0.1	neg.	neg.	278	***12.6***	***3.09***	0.01	***1.83***	***1.27***	***3.33***	0.00
14	n.d.	0.01	neg.	22	n.d.	***11.9***	***1.08***	n.d.	***12.1***	0.00	0.00
15	n.d.	0.1	0.01	33.1	n.d.	***0.79***	***0.58***	n.d.	**0.20**	***0.88***	0.03
16	n.d.	0.01	0.0001	60	n.d.	***1.45***	***16.2***	n.d.	**0.23**	***0.43***	***1.39***
17	n.d.	0.0001	0.0001	91.6	n.d.	***19.4***	***7.52***	n.d.	***1.82***	***12.7***	**0.31**
18	n.d.	0.01	0.1	35.4	n.d.	***0.46***	***0.73***	n.d.	**0.18**	**0.31**	**0.10**
19	n.d.	0.001	0.1	202	n.d.	***34.2***	***0.59***	n.d.	***34.2***	***7.90***	**0.22**
20	n.d.	0.1	0.1	740	n.d.	***3.26***	***1.48***	n.d.	***2.66***	***0.94***	0.01
21	n.d.	0.001	0.0001	47	n.d.	***0.94***	***15.7***	n.d.	***1.90***	0.02	***3.66***
22	n.d.	neg.	0.001	21.7	n.d.	<0.1	***6.60***	n.d.	0.01	0.03	***2.50***
23	n.d.	0.001	neg.	197	n.d.	***9.30***	***0.43***	n.d.	***6.72***	***4.71***	0.02
24	n.d.	0.1	0.001	41.2	n.d.	***1.61***	***12.6***	n.d.	***0.55***	***6.07***	***4.66***
25	n.d.	0.01	0.1	181	n.d.	***2.52***	***0.71***	n.d.	***7.80***	0.06	0.06
26	n.d.	0.0001	neg.	68.6	n.d.	***14.0***	***0.58***	n.d.	***7.56***	0.08	0.01
27	n.d.	neg.	0.0001	568	n.d.	**0.12**	***>100***	n.d.	0.07	0.04	***>100***
28	n.d.	0.0001	0.0001	65.8	n.d.	***1.29***	***3.29***	n.d.	***1.66***	0.02	***0.66***

Patients with a systemic reaction after an insect sting (grade I to IV according to Ring and Messmer^46^) were included for BAT. Patients 1 to 13 are from Spain (from the area of Barcelona) and allergic to PDV and/or YJV and patients 14 to 28 are from Germany (from the area of Munich) and allergic to YJV and/or HBV. sIgE and tIgE levels were determined using the UniCAP 250 system (Thermo Fisher Scientific). bold/italic: sIgE ≥ 0.35 kU_A_/L; bold: sIgE between 0.1 and 0.35 kU_A_/L.

n.d., not determined; neg., negative.

^1^For intradermal skin tests the lowest venom concentration [µg/mL] that gave a positive result is displayed.

^2^Measured with ImmunoCAP-ISAC (Thermo Fisher Scientific).

In order to address cross-reactivity of Pol d 3 in BAT, 15 patients from Germany (from the area of Munich) with allergy to YJV and/or HBV were analysed for their reactivity with Pol d 3, Api m 5 and Ves v 3 (Fig. 4b). Five patients showed basophil activation by Pol d 3 (patients 15, 16, 18, 19, 28) six by Api m 5 (patients 15, 17, 18, 21, 27, 28) and seven by Ves v 3 (15, 18, 19, 20, 24, 26, 28). Again, in most cases activation patterns by Api m 5 and Ves v 3 match sIgE and skin test data, indicating allergy to HBV, YJV or both (Table 1). Three of the Pol d 3-reactive patients, showed basophil activation by all three allergens (patients 15, 18, 28) and one by Pol d 3 and Ves v 3 (patient 19). Only patient 16 showed exclusive basophil activation by Pol d 3, but the overall activation (including the positive control) was very low.

### sIgE-reactivity of Pol d 3

**Figure 5:**
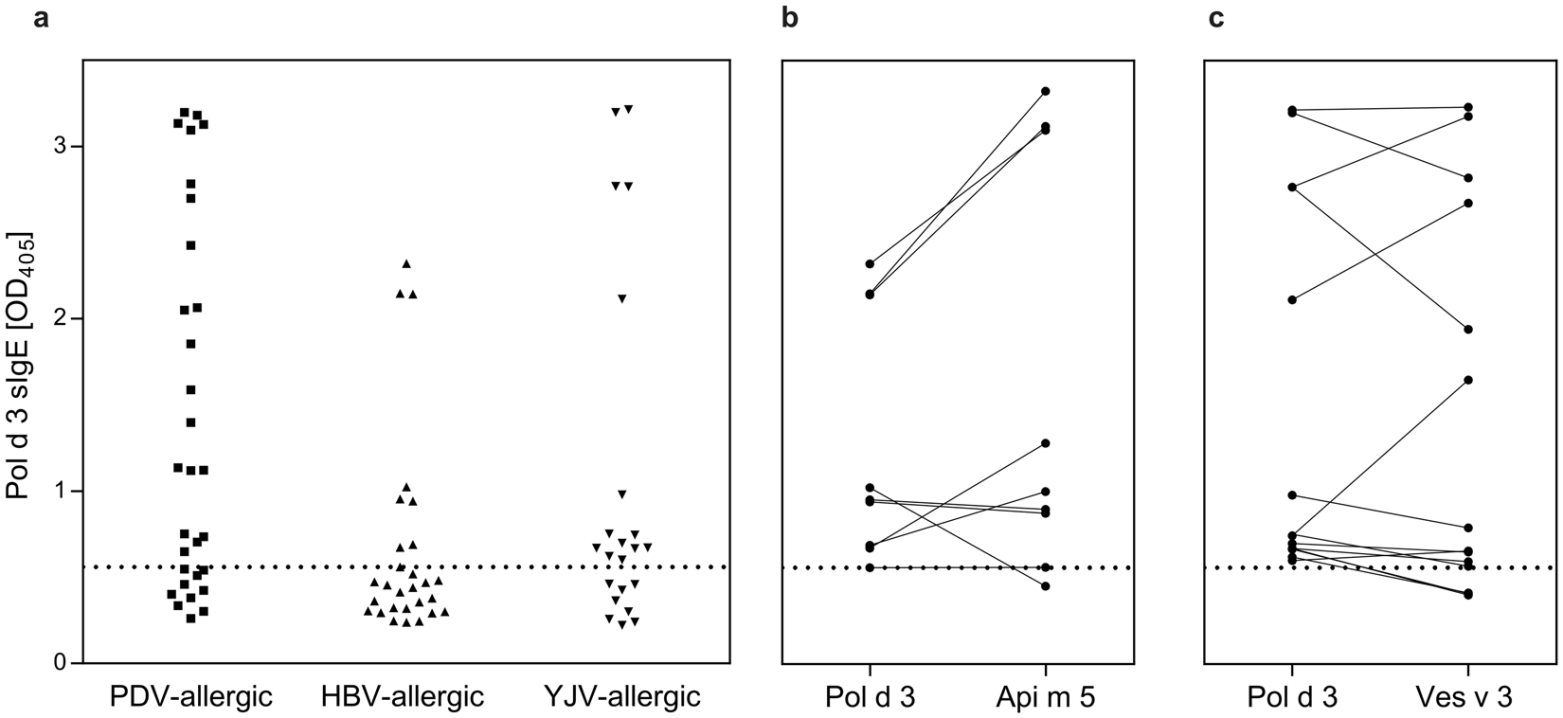
sIgE reactivity of individual hymenoptera venom-allergic patients with recombinant DPP IV allergens in ELISA. (**a**) sIgE immunoreactivity of PDV- (n = 30), HBV- (n = 28) and YJV-allergic patients (n = 20) with Pol d 3. (**b**) Comparative sIgE immunoreactivity of Pol d 3-positive HBV-allergic patients with Pol d 3 and Api m 5. (**c**) Comparative sIgE immunoreactivity of Pol d 3-positive YJV-allergic patients with Pol d 3 and Ves v 3. The lower end cut-off of the ELISAs is represented by dotted lines.

The sIgE to the newly identified Pol d 3 was addressed by ELISA. 24/30 of the patients from Spain (from the area of Cordoba) were diagnosed with PDV allergy by a combination of clinical history, skin test and sIgE levels to venom extracts and allergen components (Supplementary Table S1). For the remaining 6 patients (10, 11, 15, 23, 26, 30) diagnostic results were less clear and, hence, allergy to PDV and YJV cannot be excluded. In the group of Spanish patients 20/30 (66.7%) exhibited pronounced sIgE-reactivity with Pol d 3 (Fig. 5a).

In order to address sIgE cross-reactivity of Pol d 3 in HBV- and YJV-allergic patients from Germany (from the area of Munich), reactivity with the recombinant allergen was assessed. The patient groups were selected either for primary sensitization to HBV or YJV by detailed clinical characterisation (Supplementary Table S1). Nevertheless, in these groups allergy to both species cannot be fully excluded. However, since PDV allergy in Germany is virtually not present, primary sensitization to PDV can be excluded with high probability. 9/28 (32.1%) of HBV- and 14/22 (63%) of YJV-allergic patients showed reactivity with Pol d 3, respectively (Fig. 5a). Interestingly, most of the HBV- and YJV-allergic patients with sIgE to Pol d 3 also exhibited sIgE to the homologous allergens from HBV (Api m 5) (Fig. 5b) and YJV (Ves v 3) (Fig. 5c), respectively. Only for one patient with HBV allergy and for 3 YJV-allergic patients with very weak reactivity to Pol d 3, reactivity with the homologous allergens was slightly below the cut-off of the ELISA.

## Discussion

*Polistes dominula* is one of the main elicitors of hymenoptera venom allergy in Southern Europe as well as in parts of the United States. Moreover, *Polistes dominula* is a very invasive species spreading from the warmer to the more moderate climate zones and is therefore likely to gain importance also in other areas. However, compared to other species such as *Apis mellifera* or *Vepula vulgaris*, the knowledge of the composition of important PDV allergens on a molecular level is restricted. Additionally, to date the availability of diagnostic tools for the discrimination between PDV and YJV allergy is very limited. Therefore, our study aimed to identify and immunologically characterise novel important allergens of PDV to extend the available repertoire of venom allergens for analyses on a molecular level.

In this study, we were able to identify the sIgE-reactive 100 kDa allergen of PDV as dipeptidyl peptidase IV and homologue of the well-established DPP IV allergens Api m 5 and Ves v 3 of HBV and YJV, which were shown to represent relevant allergens of clinical importance^18,29,39^. Protein sequence identity with the corresponding allergens of HBV and YJV is 54% and 76.1%, respectively. Due to its allergenic properties and homology to the YJV allergen Ves v 3 the new PDV allergen was assigned as Pol d 3.1010 to the IUIS/WHO allergen nomenclature database^34^.

In order to immunologically characterise Pol d 3, the protein was recombinantly produced as soluble and properly folded protein in Sf9 insect cells. Lectin-staining confirmed that Pol d 3 is a glycoprotein, a fact that explains the difference between the calculated molecular mass of the polypeptide chain of 86.2 kDa and the apparent molecular mass of approximately 100 kDa observed in SDS-PAGE and immunoblot analyses. However, recombinant Pol d 3 was devoid of CCD-reactivity as shown previously for other allergens produced in Sf9 insect cells^29,35–37^. Intriguingly, the reactivity of Pol d 3 with a polyclonal Api m 5-specific antiserum^40^ hinted to a pronounced protein-based cross-reactivity of the DPP IV allergens of the different hymenoptera species. This cross-reactivity was further confirmed by the reactivity of a serum pool from Pol d 3-reactive PDV-allergic patients with the DPP IV allergens Api m 5 and Ves v 3.

To access the relevance of Pol d 3 as allergen, sIgE-reactivity of PDV-allergic patients with this newly identified PDV component was addressed. Thereby, over 66% of PDV-allergic patients exhibited pronounced sIgE to Pol d 3. This is in a comparable range found for the reactivity of either HBV- or YJV-allergic patients with the corresponding DPP IV allergens Api m 5 and Ves v 3, respectively. In previous studies, it was demonstrated that 58.3% to 61.7% of HBV-allergic patients and 57% of YJV-allergic patients show sIgE to Api m 5 and Ves v 3, respectively^18,29,39^. These data clearly suggest that Pol d 3 represents a major allergen of PDV. As *Polistes dominula* and *Vespula vulgaris* coexist in Spain and are difficult to discriminate, systemic reactions due to both insects cannot be excluded in the Spanish patient population.

Additionally, 32% of HBV- and 63% of YJV-allergic patients exhibited sIgE to the new PDV allergen. The majority of these patients additionally showed reactivity with the homologous allergen of HBV (Api m 5) or YJV (Ves v 3), indicating that the sIgE to Pol d 3 is a result of extensive protein-based cross-reactivity. Moreover, the lower degree of cross-reactivity between Pol d 3 and Api m 5 compared to Pol d 3 and Ves v 3 most likely reflects the lower sequence identity between the PDV and HBV allergen and, thus, of less conserved IgE epitopes.

So far phospholipases A1 (Pol d 1 and Ves v 1) and antigens 5 (Pol d 5 and Ves v 5) are well established cross-reactive allergen pairs of PDV and YJV^2,3,28,41^. Although it is likely that also the hyaluronidases of PDV and YJV are cross-reactive, no reliable data exist. Moreover, at least YJV hyaluronidase seems to be of limited clinical relevance^37,42^. In this study DPP IV allergens were identified as novel pair of cross-reactive major allergens, responsible for the frequently observed double-positive sIgE test results with PDV and YJV. Additionally, our analyses provide for the first time a molecular basis for the observed cross-reactivity between PDV and HBV^43^. Therefore, DPP IV allergens might be pan-allergens, present in various hymenoptera venoms. Also, the presence of DPPs IV in many snake venoms^44^ suggests functions of this enzyme class in the venoms of phylogenetically distinct species.

The capacity of Pol d 3 to activate effector cells and to initiate an allergic response was assessed by basophil activation testing and proved its role as potent allergen. In the Spanish patient group the basophil activation patterns by Pol d 3 and Ves v 3 matched the data of skin testing and sIgE levels to allergen extracts and components. For the German patients, this was also the case for the basophil activation data obtained with Api m 5 and Ves v 3. However, in this patient group Pol d 3 clearly demonstrated to be cross-reactive also in BAT. For these patients PDV allergy can be excluded with high probability since it is virtually not present in Germany. Therefore, Pol d 3-reactivity in BAT in these patients most likely is due to cross-reactivity. This is further supported by the fact that most Pol d 3-reactive patients additionally show basophil activation in response to Ves v 3 (and Api m 5). Only one patient showed weak basophil activation in response to Pol d 3 only but also very low general activation according to the positive control. However, if these results are of clinical relevance is difficult to determine since diagnostic sting provocation testing is ethically not justifiable. Nevertheless, the data demonstrate that BATs with recombinant cross-reactive major allergens represent a helpful tool to identify the allergy-eliciting venom as shown previously^28^.

To date, in clinical practice the discrimination between allergy to PDV and YJV is quite challenging. In contrast, for the discrimination between allergy to YJV and HBV an extended component-resolved diagnostics has evolved and has shown to provide added benefit for clinical decisions^4^. It was proposed that PDV and YJV allergy should be discriminated by measurement of the level of sIgE to phospholipases A1 (Pol d 1 and Ves v 1) and antigens 5 (Pol d 5 and Ves v 5)^3^. However, in addition to the YJV allergens, so far only Pol d 5 is available for routine diagnostics. In a former study, we were able to demonstrate that for most of the patients, for whom the allergy-relevant venom was clearly identified, the sIgE level with the appropriate antigen 5 (Pol d 5 or Ves v 5) indeed was higher^28^. However, this does not hold true for all patients. Moreover, for many patients the sIgE levels are in a very comparable range, hence, results will be difficult to interpret in many cases.

Of course, it would be of major interest to identify species-specific marker allergens that would allow a reliable and easy discrimination between PDV and YJV allergy. However, all identified major allergens of the two venoms, including phospholipases A1, antigens 5 and dipeptidyl peptidases IV, exhibit a high degree of cross-reactivity. Hence, the development of a component-resolved diagnostic approach, comparable to that for the discrimination between HBV and YJV allergy is difficult to realize, in our opinion. Nevertheless, the extension of the available panel of allergen components also for routine diagnostics of PDV (and YJV) allergy will generate added value for advanced diagnostics. Certainly, the combination of the results of sIgE measurements to more than one major allergen will help to create a clearer diagnostic picture and to facilitate diagnostic decisions also in vespid venom allergy. Therefore, the newly identified major allergen of PDV, Pol d 3, together with its counterparts of YJV and HBV, might become a key element for molecular diagnostics of hymenoptera venom allergy. Moreover, the detailed knowledge of the allergen composition of different insect venoms will help to understand the immunological mechanisms of venom allergy and therapeutic outcome.

## Methods

### Patients

Blood and/or sera of 108 patients with allergy either to PDV, HBV and/or YJV were analysed. 65 patients were from the area of South Bavaria (Munich, Germany), 30 patients were from the area of Córdoba, Spain and 13 patients were from Barcelona, Spain. As PDV allergy is almost not present in Germany, allergic reactions to this species can be excluded with high probability and the German patients were allergic to YJV and/or HBV. Patients from Córdoba were primarily allergic to PDV and patients from Barcelona were allergic to PDV and/or YJV. As *Polistes dominula* and *Vespula vulgaris* coexist in Spain and are difficult to discriminate, systemic reactions due to both insects cannot be excluded.

The diagnosis of venom allergy was based on a combination of clinical history of an allergic sting reaction, a positive intradermal skin test, and/or positive sIgE levels to PDV, YJV and/or HBV (i77, i3, i1) and allergen components (i208, i209, i210, i211) (UniCAP250; Thermo Fisher Scientific, Uppsala, Sweden).

All patients had given informed written consent to draw additional blood samples. The study was approved by the ethics committee of the Faculty of Medicine of the Technical University of Munich (protocol number 5478/12), the ethics committee for clinical research of Reina Sofía University Hospital Cordoba (protocol number BLA-VIT-2015-01) and the ethical committee for clinical investigation of the Hospital Clinic of Barcelona (protocol number 2011/6605). All patients were recruited from clinical routine and the obtained data are not part of another study reported elsewhere. All experiments were performed in accordance with relevant guidelines and regulations.

### Protein biochemistry

Pol d 3 was identified by tandem mass spectrometry analyses on a MALDI-TOF instrument. A detailed description is given in the Supplementary Methods.

### Cloning and recombinant production of venom dipeptidyl peptidases IV

The coding region of Pol d 3 was amplified from *Polistes dominula* venom gland cDNA and plasmids coding for Api m 5 and Ves v 3 were generated as described previously^29,45^. Cloning and recombinant production in *Spodoptera frugiperda* (Sf9) insect cells is described in detail in the Supplementary Methods.

### Immunoreactivity of patient sera with recombinant dipeptidyl peptidases IV

sIgE immunoreactivity of sera with the recombinant venom DPPs IV was assessed by ELISA. A detailed description of the ELISA is given in the Supplementary Methods. The lower end functional cut-off, indicated as dotted lines, was calculated as the mean of the negative controls summed with 3 times the standard deviation (SD) of the mean and additionally 10% of the resulting value.

### Basophil activation test

Basophil activation tests were performed in 15 YJV- and/or HBV-allergic patients, and in 13 PDV- and/or YJV-allergic patients as described previously^38^, using the Flow CAST (Bühlmann Laboratories AG, Schönenbuch, Switzerland). Allergen concentrations were 2, 10, 50 and 250 and 1000 ng/mL. A detailed description is given in the Supplementary Methods.

### Other methods

SDS-PAGE, Western blotting, DPP IV activity and CD spectroscopy are described in the Supplementary Methods.

### Data availability

All data generated or analysed during this study are included in this published article and its Supplementary Information files.

## Electronic supplementary material

Supplementary Information
