# Supplementary material for: The high molecular weight dipeptidyl peptidase IV Pol d 3 is a major allergen of Polistes dominula venom
Source: Sci Rep. 2018 Jan 22;8:1318. doi: 10.1038/s41598-018-19666-7 (PMC5778000; doi:10.1038/s41598-018-19666-7)
Supplement: Supplementary file 1 — Supplementary Information [file 41598_2018_19666_MOESM1_ESM.pdf]

The high molecular weight dipeptidyl peptidase IV Pol d 3 is a major allergen of *Polistes dominula* venom

Maximilian Schiener, Christiane Hilger, Bernadette Eberlein, Mariona Pascal, Annette Kuehn, Dominique Revets, Sébastien Planchon, Gunilla Pietsch, Pilar Serrano, Carmen Moreno-Aguilar, Federico de la Roca, Tilo Biedermann[3], Ulf Darsow, Carsten B. Schmidt-Weber, Markus Ollert, Simon Blank

Supplementary Methods

Protein biochemistry – MALDI-TOF

Gel pieces from the 100 kDa band of *Polistes dominula* venom (Entomon, Firenze, Italy) were excised from the SDS-PAGE and transferred to 0.65 µL microfuge siliconized tubes (Sigma Aldrich, USA). Gels plugs were washed twice for 10 min with ultrapure water (Biosolve, The Netherlands) and equilibrated in buffer (NH_4HCO_3, 50 mM, pH 8) for 10 min. The buffer was removed and 20 µL of pure acetonitrile (Biosolve, The Netherlands) were added to the gel pieces until they became white. The acetonitrile is discarded and the gel particles were dried with the help of a SpeedVac.

Two microliters of trypsin solution (12.5 ng/µL, 50 mM NH_4HCO_3, pH 8) were incorporated into the gel particles by passive rehydration during 45 minutes on ice. Finally, 8 µL of 50 mM NH_4HCO_3 pH 8 were added to cover gel particles. The samples were incubated 4 hours at 37°C. Following the enzymatic digestion, gel particles were incubated in 10 mM dithiotreitol (50 mM NH_4HCO_3 pH8) for 20 min at 56°C.

Of the digestions, 0.5 µL were spotted on a MALDI target plate (Polished steel 384 MALDI target plate, Bruker, Germany) and 0.3 µL of matrices solution was added (25 mg/mL alpha-cyano-4-hydroxycinnamic acid and 4 mg/mL 2,5-dihydroxybenoiquem in 50% Acetonitrile containing 0.1% TFA) (Bruker, Germany) according to dried-droplet method. An external calibration was done before each analysis with trypsin digested bovine serum albumin according to manufacturer's instructions (MALDI-TOF Ultraflex I, Bruker, Germany). From the protein mass fingerprints, 5 peptides were selected to generate fragmentation spectra (collision-induce dissociation). Of the digestions, 0.7 µL were spotted on a MALDI target plate (384 MALDI target plate, AB Sciex, USA) and 0.7 µL of matrix solution was added (7 mg/mL alpha-cyano-4-hydroxycinnamic acid in 50% Acetonitrile containing 0.1% TFA) according to dried-droplet method. Fragmentation spectra were

acquired via collision-induce dissociation on a Sciex TOF-TOF 5800 mass spectrometer. The sequences were deduced by manual *de novo* sequencing.

Cloning of venom dipeptidyl peptidases IV

mRNA of *Polistes dominula* was isolated by disruption of two venom glands with the Quiagen TissueLyser LT in 1 mL TriFast buffer (Peqlab/VWR, Erlangen, Germany) and subsequent RNA precipitation. Obtained mRNA was cleared from genomic DNA contamination by DNAse treatment on RNeasy Mini Spin Columns (Quiagen, Hilden, Germany). cDNA was synthesized from mRNA using an oligo-dT-primer and SuperScriptIII reverse transcriptase (Invitrogen/Thermo Fisher Scientific, Schwerte, Germany). Pol d 3 was amplified from venom gland cDNA by PCR (Q5, NEB, Frankfurt am Main, Germany) without signal peptide sequence using primers producing restriction enzyme recognition sites to be able to digest with NheI and NotI (forward primer: GAT C**GC TAG C**TA TGT CCT CGA TAA G; reverse primer: GAT C**GC GGC CGC** TCA GTG AGT ATT AGA CCA ACT GAA; restriction sites in bold). PCR products were cloned via NheI/XbaI and NotI into the digested baculovirus transfer vector pAcGP67-B (BD Pharmingen, Heidelberg, Germany) containing an N-terminal 10-fold His-tag and V5-epitope tag. pAcGP67-B plasmids coding for Api m 5 and Ves v 3 were generated as described previously[1].

Recombinant baculovirus production and expression in baculovirus-infected insect cells

Proteins were expressed as secreted full-length proteins in *Spodoptera frugiperda* (Sf9) insect cells (Thermo Fisher Scientific, Schwerte, Germany) by infection with recombinant baculovirus as described before[1,2]. In brief, Sf9 cells were grown at 27 °C in serum-free medium (Express Five SFM; Lonza, Verviers, Belgium) containing 10 µg/mL gentamicin (Sigma, Taufkirchen, Germany). Recombinant baculoviruses were generated by

co-transfection of Sf9 cells with the resulting pAcGP67-B vectors containing the dipeptidyl peptidases IV and ProGreen^{TM}-Baculovirus DNA (Ab Vector, San Diego, USA). Two or three rounds of virus amplification produced high titer virus stocks. High titer stocks were used to infect 400 mL Sf9 suspension cultures ($1.5 \cdot 10^6$ cells/mL) in 2000 mL flasks. For protein expression, infected cells were cultured at 27 °C and 110 rpm for 72 h.

For protein purification, the supernatant was collected and cleared from cells and debris by centrifugation at 4000 xg and 4° C and applied to a nickel-chelating affinity matrix (HisTrap excel, GE Healthcare Life Sciences, Freiburg, Germany). The column was washed with phosphate buffered saline (PBS, pH 8; 1 mM KH_2PO_4, 155 mM NaCl, 3 mM Na_2HPO_4 x $7H_2O$). Unspecific bound proteins were pre-eluted with PBS containing 45 mM imidazole and the recombinant proteins were eluted with PBS containing 300 mM imidazole. SDS-PAGE and Western blotting confirmed purity of the proteins.

Immunoreactivity of patient sera with recombinant dipeptidyl peptidases IV

To assess specific IgE immunoreactivity of human sera, 384-well microtiter plates (Nunc, Thermo Fisher Scientific, Ulm, Germany) were coated with 50 µg/mL purified recombinant DPPs IV in PBS at 4 °C overnight and blocked with 40 mg/mL nonfat dry milk powder (AppliChem, Darmstadt, Germany) in PBS at RT for 1h. Human sera were diluted 1:2 with PBS and a final volume of 20 µL was added to the recombinant proteins and incubated overnight at 4 °C. After washing 4 times with PBS, bound IgE was detected with a 1:1000 dilution of a monoclonal alkaline phosphatase-conjugated anti-human IgE antibody (BD Pharmingen, Heidelberg, Germany). Subsequently, after washing 4 times with PBS, 50 µL of substrate solution (5 mg/mL 4-nitrophenylphosphate; AppliChem, Darmstadt, Germany) was added per well and the absorbance was read at 405 nm. The lower end functional cut-off, indicated as dotted lines, was calculated as the mean of the negative

controls summed with 3 times the standard deviations (SD) of the mean and additionally 10% of the resulting value.

Basophil activation test

Basophil activation tests were performed as described previously[3], using the Flow CAST (Bühlmann Laboratories AG, Schönenbuch, Switzerland).

In short, venous blood was collected in 10 mL EDTA tubes (1.6 mg EDTA/mL blood) and stored at 4 °C. For each patient and allergen, polystyrene tubes were prepared with 50µl of allergen in different concentrations (2, 10, 50, 250 and 1000 ng/mL) diluted in stimulation buffer. A monoclonal anti-FcεRI antibody served as positive control and stimulation buffer alone was used as negative control. Subsequently, 100 µL stimulation buffer containing calcium, heparin and IL-3 (2 ng/mL), 50 µL blood and 20 µL staining reagent (anti-CD63-fluorescein isothiocyanate and anti-CCR3-phycoerythrin monoclonal antibodies) were added to the antigen dilutions and incubated at 37 °C for 25 minutes. Adding 2 mL lysis buffer for 5 min at RT stopped stimulation. After centrifugation for 5 min at 500 x g, the supernatant was decanted and 300 µL washing buffer were added to each tube.

Cells were analysed by flow cytometry. Basophilic cells were selected out of the lymphocyte population using anti-CCR3 and the upregulation of the activation marker CD63 was calculated by the percentage of the $CD63^+$ of total basophilic cells. The cut-off, represented as dotted line, was set to 10% $CD63^+$ cells as recommended by the supplier.

SDS-PAGE and Western Blotting

For purity analysis and immunoblot procedures, the purified recombinant allergens or crude hymenoptera venom were separated by homemade 10% SDS-PAGE gels (recombinant allergens) or AnykDTM Mini-Protean® TGX gels (Bio-Rad, München, Germany) (*Polistes*

dominula venom) and stained with either Coomassie Brilliant Blue G-250 (AppliChem, Darmstadt, Germany) or SyproRuby staining (ThermoFischer Scientific). For immunoblot procedures proteins were immobilized onto nitrocellulose membranes. Blot membranes were blocked, incubated with mouse monoclonal anti-V5 antibody (Thermo Fisher Scientific, Schwerte, Germany), biotinylated *Galanthus nivalis* agglutinin (Vector Laboratories, Peterborough, United Kingdom), rabbit polyclonal anti-HRP antiserum (Thermo Scientific, Schwerte, Germany), rabbit polyclonal anti-Api m 5 antiserum (Davids Biotechnology, Regensburg, Germany)[4] or a pool of sera of allergic individuals and detected with the alkaline phosphatase conjugates of anti-human IgE (BD Bioscience,), anti-rabbit IgG, anti-mouse IgG or ExtrAvidin (Sigma, Taufkirchen, Germany). The bound secondary antibodies were visualized with nitrotetrazolium blue chloride and 5-bromo-4-chloro-3-indolyl phosphate (AppliChem, Darmstadt, Germany) as substrate.

Enzymatic activity of recombinant Pol d 3

The wells of an untreated 96-well plate were filled with 50 µL of a 1 mM solution of the synthetic DPP IV substrate Gly-Pro-ρ-nitroanilide HCl (Sigma, Taufkirchen, Germany) in PBS. For inhibition experiments serial dilutions (1.5 to 300 µM) of the inhibitor Diprotin A (Ile-Pro-Ile, Sigma, Taufkirchen, Germany) were added. Of recombinant Pol d 3, 2.5 µg in 50 µL PBS was added to each well and absorbance was read at 405 nm at intervals of 2.5 min for 60 min at 37 °C.

CD spectroscopy

Circular dichroism spectra were recorded at 20°C using a Chirascan CD spectrometer (Applied Photophysics, Leatherhead, UK). A 0.5-mm optical path length quartz cell was used to obtain spectra in the far-UV region (190 to 260 nm) at a protein concentration of 6µM in

20 mM TRIS-HCl, pH 8. The CD spectra were acquired at a scan speed of 1 s/nm and a step resolution of 1 nm. Spectra were measured in triplicate and averaged.

Supplementary Figure S1

DPP IV activity of Pol d 3. Dipeptidyl peptidase IV activity of recombinant Pol d 3 by (a) cleavage of the chromogenic substrate glycine-proline-ρ-nitroanilide (replicates shown with standard deviation) and (b) inhibition of activity by the specific DPP IV inhibitor Diprotin A (Ile-Pro-Ile).

a

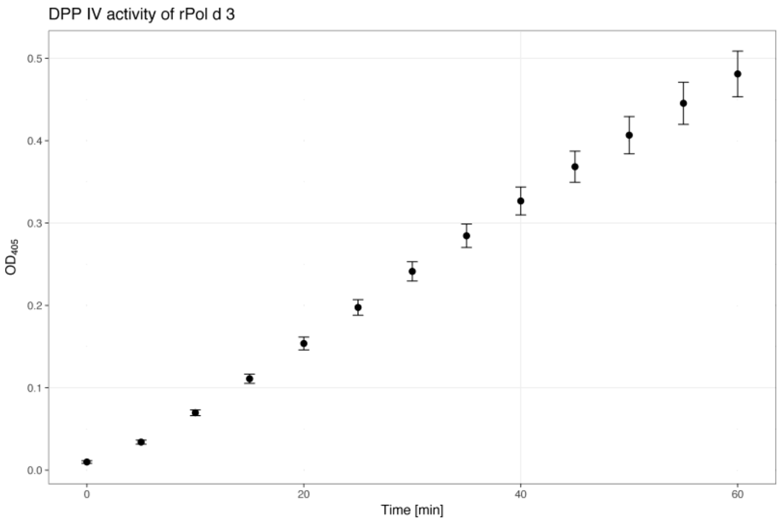

b

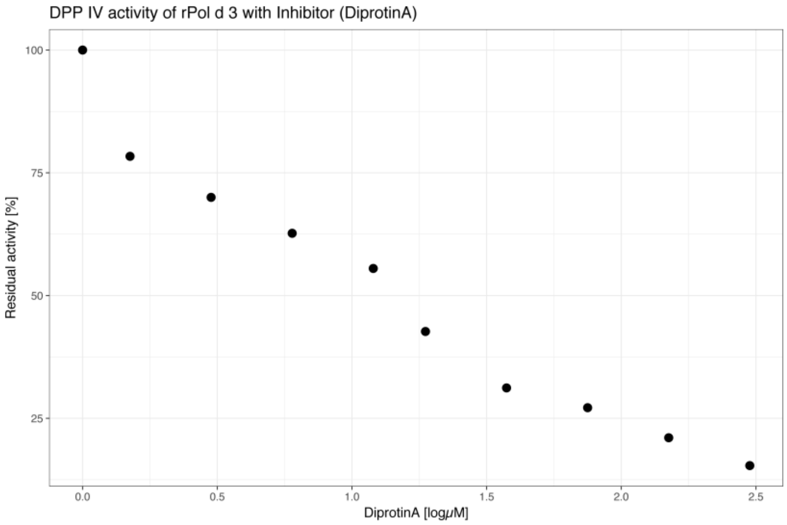

Supplementary Figure S2

Circular dichroism of recombinant Pol d 3. The structural features of recombinant Pol d 3 were assessed by CD spectroscopy and the structure was found to be dominated by β sheets. Moreover, the obtained experimental data fit very well to the structural elements predicted by the CDSSTR method[5,6].

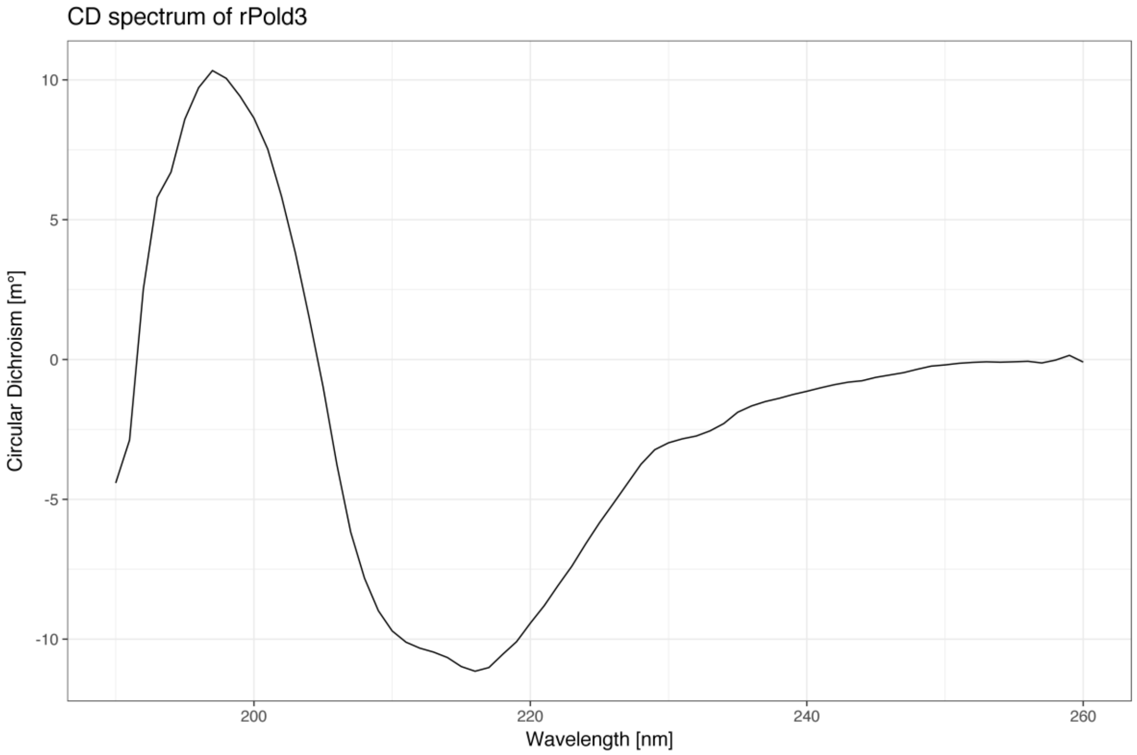

Supplementary Figure S3

Full-length gels and blots of Fig. 1 and Fig. 3. (a) Full-length gel and blot of Fig. 1. SDS-PAGE and protein staining (SyproRuby staining) of PDV (left) and sIgE-immunoreactivity of pooled sera from PDV-allergic patients with PDV in Western blot (right). (b) Full-length gel and blot of Fig. 3. SDS-PAGE and Western blot analyses of Pol d 3 recombinantly produced in Sf9 in comparison with the HBV and YJV homologues Api m 5 and Ves v 3 either by Coomassie blue staining or anti-V5 epitope antibody, GNA (*Galanthus nivalis* agglutinin), anti-HRP antiserum, anti-Api m 5 antiserum and pooled sera of PDV-allergic patients. For anti-V5 epitope detection Pol d 3/Api m 5 and Ves v 3 and for detection with polled sera all three allergens were analyzed on separate blots.

a

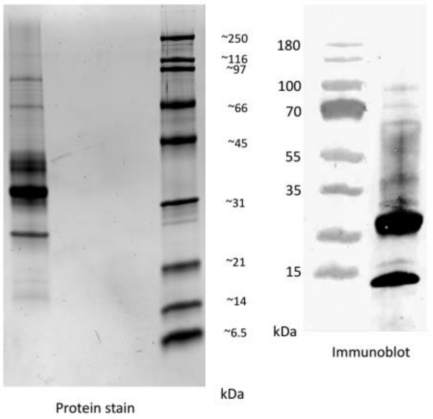

b

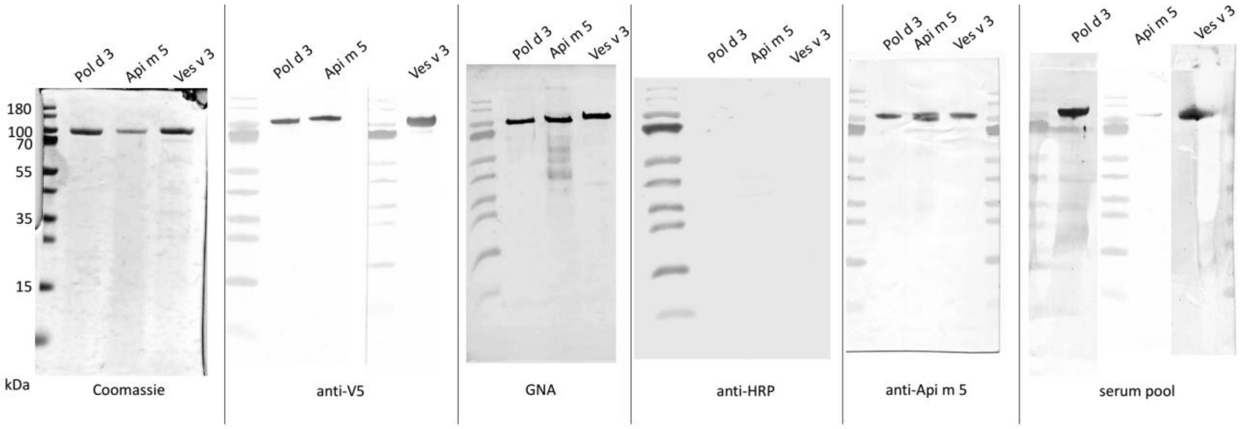

Supplementary Table S1

Clinical and serological data of patients assessed in sIgE reactivity analysis

The sIgE levels to *Polistes dominula* venom (PDV) (i77), yellow jacket venom (YJV) (i3), honeybee venom (i1), Pol d 5 (i210), Ves v 5 (i209), and Api m 1 (i208) as well as the total IgE levels were determined with the UniCAP250 system (Thermo Fisher Scientific, Uppsala, Sweden). The sIgE levels to the recombinant DPP IV allergens Pol 3, Ves v 3 and Api m 5 were determined by ELISA as described in the methods section. sIgE levels above the lower end cut-off are displayed in red. For intradermal testing of patients, serial 10-fold dilutions of venom extracts with concentrations ranging from 0.0001 to 0.1 mg/L were performed. Intradermal tests were rated positive when the wheal size was >5 mm in diameter with a surrounding erythema. Shown in the table is the lowest venom extract concentration that gave a positive result in intradermal skin testing. Systemic sting reactions were graded according to Ring and Messmer[7].

PDV-allergic patients from the area of Cordoba in Spain:

Patient ID	Sting reaction grade	Skin test (i.c.) PDV [µg/mL]	Skin test (i.c.) YJV [µg/mL]	tIgE [kU/L]	sIgE PDV (i77) [kU$_A$/L]	sIgE YJV (i3) [kU$_A$/L]	sIgE Pol d 5 (i210) [kU$_A$/L	sIgE Ves v 5 (i209) [kU$_A$/L]	sIgE Pol d 3 [OD$_{405}$]
1	II	0.1	neg.	47	n.d.	n.d.	3.34	1.15	3.180
2	I	0.001	neg.	n.d.	78.6	7.64	4.96	0.76	3.094
3	II	0.1	neg.	n.d.	2.63	0.67	n.d.	n.d.	0.425
4	II	0.1	neg.	n.d.	4.00	<0.35	n.d.	n.d.	0.459
5	III	0.01	neg.	n.d.	4.71	0.56	2.96	0.40	0.705
6	I2	0.1	0.1	281	5.58	1.41	2.76	2.01	2.425
7	I	0.001	neg.	258	4.04	0.19	0.57	0.05	3.134
8	II	0.01	neg.	142	8.60	0.00	0.00	0.00	1.589
9	II	0.1	neg.	n.d.	8.59	0.00	6.61	0.00	0.260
10	II	0.1	0.1	n.d.	9.04	18.5	12.2	19.5	1.398
11	II	0.1	0.1	n.d.	19.7	11.9	0.51	0.23	2.697
12	III	neg.	neg.	9.	7.02	0.04	6.68	0.04	0.381
13	III	0.01	neg.	159	18.5	5.17	0.99	0.55	2.048
14	I	0.1	neg.	n.d.	27.2	1.11	0.05	0.02	1.853
15	II	0.1	0.001	n.d.	n.d.	13.0	n.d.	n.d.	0.751
16	I	0.01	neg.	29	1.61	1.27	n.d.	n.d.	0.402
17	III	0.1	neg.	72.40	2.67	0.49	n.d.	n.d.	0.512
18	III	0.1	neg.	3410	3.93	1.13	n.d.	n.d.	0.736
19	I	0.01	neg.	674	22.0	6.60	n.d.	n.d.	3.127
20	II	0.001	neg.	231	116	4.89	3.68	6.33	0.648
21	III	0.1	neg.	n.d.	2.80	0.00	0.00	0.00	0.548
22	I	0.01	0.01	32	2.17	3.26	5.69	4.71	1.122
23	II	0.01	0.01	102	16.6	11.7	12.7	9.90	2.783
24	III	0.01	0.1	n.d.	74.7	39.2	n.d.	n.d.	1.120
25	I	0.01	neg.	n.d.	4.54	2.79	n.d.	n.d.	0.542

26	II	0.1	0.1	n.d.	95.2	86.0	91.1	129	3.197
27	III	0.01	neg.	n.d.	7.92	0.47	12.7	2.41	1.136
28	II	neg.	neg.	n.d.	2.10	0.00	1.97	0.00	0.301
29	I	0.01	0.1	n.d.	68.3	28.4	0.00	0.00	2.063
30	II	neg.	neg.	n.d.	0.82	0.70	1.60	1.84	0.335

YJV-allergic patients from the area of South Bavaria (Munich) in Germany:

Patient ID	Sting reaction grade	Skin test (i.c.) YJV [µg/mL]	tIgE [kU/L]	sIgE YJV (i3) [kU_A/L]	sIgE HBV (i1) [kU_A/L]	sIgE Ves v 5 (i209) [kU_A/L]	sIgE Api m 1 (i208) [kU_A/L]	sIgE Pol d 3 [OD_{405}]	sIgE Ves v 3 [OD_{405}]
1	III	0.001	120	35.8	0.86	39.2	0.49	0.980	0.792
2	II	0.0001	1491	26.4	0.22	53.3	0.06	0.672	0.596
3	II	0.0001	1847	>100	>100	>100	0.89	2.767	3.177
4	II	0.0001	64.2	7.56	0.02	12.0	0.02	0.667	0.410
5	IV	0.01	25.5	6.91	0.50	4.47	0.01	0.699	0.651
6	I	n.d.	157	4.22	0.02	6.02	0.01	0.364	n.d.
7	II	0.01	94.8	8.53	2.09	11.3	0.34	2.765	1.940
8	III	0.0001	162	10.7	0.23	12.6	0.01	0.670	0.401
9	IV	0.001	347	26.3	6.11	11.9	1.70	0.601	0.656
10	II	n.d.	46.5	7.49	0.18	10.3	0.07	0.241	n.d.
11	III	n.d.	146	10.7	0.03	5.49	0.00	0.299	n.d.
12	II	0.0001	274	63.7	20.1	7.45	0.70	3.213	3.230
13	III	n.d.	233	32.3	6.32	32.1	0.17	0.426	n.d.
14	II	0.001	111	5.52	0.07	6.81	0.00	0.458	n.d.
15	II	n.d.	1473	13.7	0.27	4.82	0.03	0.461	n.d.
16	II	0.0001	460	21.9	3.04	23.2	0.12	0.746	1.648
17	I	0.0001	49	4.68	0.01	5.47	0.00	0.623	0.413
18	II	0.0001	107	3.55	0.01	6.87	0.00	0.222	n.d.
19	I	0.0001	12.5	4.29	0.17	3.82	0.09	0.256	n.d.
20	I	0.01	952	12.8	n.d.	67.1	n.d.	2.112	2.674
21	III	0.001	315	6.74	n.d.	7.09	n.d.	0.753	0.567
22	III	0.01	32	1.52	n.d.	1.45	n.d.	3.197	2.821

HBV-allergic patients from the area of South Bavaria (Munich) in Germany:

Patient ID	Sting reaction grade	Skin test (i.c.) HBV [µg/mL]	tIgE [kU/L]	sIgE HBV (i1) [CAP-Class]	sIgE YJV (i3) [CAP-Class]	sIgE Api m 1 (i208) [CAP-Class]	sIgE Ves v 5 (i209) [CAP-Class]	sIgE Pol d 3 [OD_{405}]	sIgE Api m 5 [OD_{405}]
1	IV	0.0001	226	4	3	3	3	2.146	3.322
2	II	0.001	222	4	2	4	1	1.025	0.453
3	II	0.01	19.8	3	0	n.d.	n.d.	0.468	n.d.
4	II	0.001	16.1	3	0	n.d.	n.d.	0.318	n.d.
5	III	0.01	150	2	0	n.d.	n.d.	0.291	n.d.
6	III	0.0001	306	5	3	n.d.	n.d.	0.954	0.897
7	II	0.01	7.56	1	0	n.d.	n.d.	0.300	n.d.
8	II	0.001	164	6	2	5	0	0.560	0.562
9	II	0.1	160	2	2	n.d.	n.d.	0.322	n.d.
10	III	0.001	7.85	2	0	2	0	0.239	n.d.
11	I	0.0001	345	5	2	n.d.	n.d.	2.319	3.097
12	II	0.01	1102	6	3	6	n.d.	0.689	1.001
13	II	0.001	67	2	0	n.d.	n.d.	0.362	n.d.
14	II	0.001	334	6	2	4	0	0.519	n.d.
15	II	0.001	78.8	2	3	0	3	0.454	n.d.
16	IV	0.001	137	4	2	3	2	0.473	n.d.
17	III	0.0001	10.3	2	0	1	0	0.305	n.d.
18	III	0.1	517	2	2	0	2	0.356	n.d.
19	II	0.0001	105	3	2	n.d.	n.d.	0.441	n.d.
20	II	0.001	38.6	3	2	3	0	2.141	3.118
21	III	0.1	419	3	0	n.d.	0	0.942	0.875
22	I	0.0001	42	4	1	0	0	0.673	1.283
23	II	0.0001	171	4	1	3	0	0.413	n.d.
24	II	0.001	34	1	n.d.	1	n.d.	0.244	n.d.
25	III	0.1	68.3	3	0	3	0	0.293	n.d.

26	III	0.1	350	1	2	1	2	0.482	n.d.
27	III	0.0001	11.5	3	0	n.d.	0	0.246	n.d.
28	III	0.001	143	2	0	n.d.	0	0.378	n.d.

HBV, honeybee venom; n.d., not determined; neg., negative; PDV, *Polistes dominula* venom; yellow jacket venom

Supplementary References

1 Blank, S. *et al.* Identification, recombinant expression, and characterization of the 100 kDa high molecular weight Hymenoptera venom allergens Api m 5 and Ves v 3. *J Immunol* **184**, 5403-5413 (2010).

2 Blank, S. *et al.* Api m 10, a genuine *A. mellifera* venom allergen, is clinically relevant but underrepresented in therapeutic extracts. *Allergy* **66**, 1322-1329 (2011).

3 Eberlein, B., Krischan, L., Darsow, U., Ollert, M. & Ring, J. Double positivity to bee and wasp venom: improved diagnostic procedure by recombinant allergen-based IgE testing and basophil activation test including data about cross-reactive carbohydrate determinants. *J Allergy Clin Immunol* **130**, 155-161 (2012).

4 Blank, S. *et al.* Component-resolved evaluation of the content of major allergens in therapeutic extracts for specific immunotherapy of honeybee venom allergy. *Hum Vaccin Immunother* **13**, 2482-2489 (2017).

5 Johnson, W. C. Analyzing protein circular dichroism spectra for accurate secondary structures. *Proteins* **35**, 307-312 (1999).

6 Sreerama, N. & Woody, R. W. Estimation of Protein Secondary Structure from Circular Dichroism Spectra: Comparison of CONTIN, SELCON, and CDSSTR Methods with an Expanded Reference Set. *Analytical Biochemistry* **287**, 252-260 (2000).

7 Ring, J. & Messmer, K. Incidence and severity of anaphylactoid reactions to colloid volume substitutes. *Lancet* **1**, 466-469 (1977).
